# Coming in hot: a qualitative investigation into perceptions of parents and doctors of reasons for the presentation of children with fever to the emergency department in England

**DOI:** 10.1136/bmjpo-2024-003039

**Published:** 2024-12-24

**Authors:** Courtney Franklin, David Taylor-Robinson, Enitan D Carrol, Paul Moran, Bernie Carter

**Affiliations:** 1 Institute of Population Health, University of Liverpool Faculty of Health and Life Sciences, Liverpool, UK; 2 Public Health and Policy, University of Liverpool, Liverpool, UK; 3 Institute of Infection and Global Health, University of Liverpool, Liverpool, UK; 4 Paediatric Infectious Diseases and Immunology, Alder Hey Children's NHS Foundation Trust, Liverpool, UK; 5 ARC NWC, NIHR, Liverpool, UK; 6 Edge Hill University Faculty of Health Social Care and Medicine, Ormskirk, UK

**Keywords:** Health services research, Child Health, Qualitative research

## Abstract

**Introduction:**

Paediatric emergency department (ED) attendances and admissions in England are increasing. Fever is a common presenting problem for these attendances. Anxiety and misperceptions surrounding appropriate management of fever persist among parents. Little evidence exists on the pathways to ED for fever, and doctors’ perceptions of why parents present their child to the ED.

**Objectives:**

To understand perceptions of parents and doctors of the reasons for ED presentation for children (0–18 years) with fever in England.

**Design:**

This forms the first part of a qualitative study, using reflective thematic analysis.

**Participants:**

15 parents (12 mothers and 3 fathers) who had taken their febrile child to hospital (2015–2023), and 5 ED doctors (4 consultants and 1 resident doctor) who had experienced treating a febrile child in an ED in England.

**Methods:**

Semistructured remote (Zoom) interviews were conducted (2022–2023).

**Results:**

Reflexive thematic analysis facilitated investigation into current parental concerns regarding fever and decision-making leading to ED attendance. The overarching theme ‘factors influencing unscheduled care’ comprised four key themes that reflected the complex interplay between factors influencing parental decision-making to seek emergency care, at the individual and wider structural level. These were parental proficiency and experience; social networks and access to services; fever phobia, uncertainty and anxiety; and reassurance. Doctors also acknowledged the importance of these factors, such as reassurance and showing compassion and further indicated a persistent educational gap surrounding fever between doctors and parents.

**Conclusions:**

We widen the evidence base of why parents attend ED for paediatric fever and their perceptions of other health services. Parents face challenges when seeking care and perceived ED as a last resort. Interventions to support parental decision-making and management of fever could help to alleviate these challenges, as well as potentially reducing the demand for emergency care.

**WHAT IS ALREADY KNOWN ON THIS TOPIC**

- Fever is a common presenting problem for emergency department (ED) attendance and is a main cause for concern for parents.
- There are many misconceptions surrounding the correct management and treatment of fever.
- The pathways taken to hospital by children with a serious infectious illness are complex and amenable to intervention.

**WHAT THIS STUDY ADDS**

- For many parents, ED is seen as the last resort for treating paediatric fever.
- Parents face many barriers when seeking appropriate primary care assessment and management of their child’s fever.
- Preventing uncertainty could help to diffuse heightened fever phobia and prevent potentially avoidable emergency hospital attendances.
- Doctors acknowledge the importance of reassurance and showing compassion, and further indicated a persistent educational gap surrounding fever between doctors and parents.

**HOW THIS STUDY MIGHT AFFECT RESEARCH, PRACTICE OR POLICY**

- The provision of consistent information resources used across different settings is crucial in informing parents and shaping their health-seeking behaviours.
- Stronger support in the community for basic home management of acute illness could strengthen parental confidence and management of fever, encouraging appropriate health-seeking behaviour.
- Acknowledging parental concern and involving parents in the decision-making process could be vital in equipping parents with correct safety netting, empowering them to manage acute illnesses at home.

## Introduction

 Previously, UK emergency department (ED) attendances increased year on year^1^ with a similar trend for admissions from the ED, posing a huge burden on the NHS financially. The 10 most common presenting problems in children account for around 85% of child ED attendances in the UK.^2 3^ Febrile illness accounts for around 14% of these consultations.^4^ There are many causes of fever, but most are due to self-limiting illness. Therefore, there remains a need to establish the reasons behind emergency presentations for febrile children.

Parental uncertainty and low risk tolerance have been previously identified as drivers for ED attendance for conditions suitable for management in less acute settings.^5^ Therefore, while increased admissions may not be attributed to increased severity of disease, increased perceived severity by parents may increase ED attendances and influence risk averse behaviours among parents and doctors.

Parental anxiety and misperceptions about appropriate management of fever persist.^6^ Despite studies of parental perceptions of fever and its management, evidence specific to England remains limited.^7^,^11^

Fragmented services can impact parents’ experiences when navigating healthcare for their child and cause confusion about where to have their health needs met.^12^,^14^ Fragmented services and parents’ problems interpreting symptoms are core modifiable factors influencing the timing of attendance and admission to hospital for children with serious infectious illness.^15^ Little evidence exists on these pathways for fever (of which many cases could be self-limiting), or doctors’ perceptions of why parents present their child to the ED and how consideration of this can affect the child’s treatment.^16^ We aimed to understand parental and doctor perceptions of the reasons for ED presentation for children with fever in England.

## Methods

### Study design

A descriptive qualitative study using semistructured remote (Zoom (Zoom Video Communications)) interviews (June 2022 to January 2023). The study followed the Consolidated Criteria for Reporting Qualitative Research guideline.^17^

### Patient and public involvement

An NIHR Applied Research Collaborative North West Coast Public Advisor (PM) was involved throughout this study, providing invaluable insight into the design, analysis and interpretation of results.

### Participants and methods

Convenience and venue-based sampling facilitated timely access of potential participants and encouraged uptake of participants from areas of higher disadvantage (the North West of England). Convenience sampling was the most accessible form of sampling and allowed all eligible, consenting members to take part in the study. Consideration was further made to incorporate a sampling frame for homogeneous convenience sampling. This has previously been done by intentionally constraining the sampling frame with respect to sociodemographic background.^18^ On advisement from our public advisor, we did not collect socioeconomic information from prospective or actual participants, as it was felt this could be perceived as intrusive and could deter participants. Instead, we used a form of venue-based sampling, identifying locations where the target population may gather, randomly selecting and visiting those locations, and systematically intercepting potential consenting participants.^19^ Recruitment was intentionally focused on Liverpool and the surrounding areas in Northwest England to capture perceptions within an area of historical and current disadvantage. This allowed sampling to be targeted to parents in areas of a higher deprivation who may have been missed using other sampling strategies. This was done by contacting nursery schools, primary schools and children’s centres to help distribute recruitment adverts, as well as focusing social media advertisement within these targeted areas.

Parents of children (0–18 years) with a presenting problem of a fever who attended an ED (January 2015–2023) in England, and doctors with experience treating a febrile child in an English ED setting, could self-refer via recruitment adverts. Virtual parent adverts were shared online (Facebook, X (formerly Twitter)) and distributed to local schools and community centres. We aimed to recruit 10–15 parents and 10–15 healthcare professionals (HCPs) as suggested for medium-sized thematic analysis studies.^20^ Despite providing an approximate sample size for this study, the adequacy of the final sample size was evaluated throughout the interview process and was open to the researcher’s ongoing interpretation. These factors were used to ensure adequate and rich data for theme development (including data saturation), and the overall sample size was determined by this, and time constraints within the research. We aimed to recruit parents with an experience after the COVID-19 lockdown measures (from 2020). However, we had some prospective parent participants showing an interest in the study, with experiences longer than this time frame. Given the slow uptake for interviews and interest of parents outside of the January 2020 criteria (but still within a time frame that would be interesting to the study), we adjusted this time frame. Flyers were distributed at community centres and a paediatric ED waiting room. Doctor adverts were distributed via social media and shared among ED clinicians.

Interview guides were developed (CF, BC and PM) using open questions (14 for parents and 7 for doctors) surrounding knowledge and management of fever, and perception of services (online supplemental file). Potential participants emailed the lead researcher (CF) who answered any questions. After gaining consent, an interview was arranged. Interviews were conducted over Zoom (Zoom Video Communications), audio recorded, transcribed and then audio recordings deleted.

### Analysis

**Figure 1:**
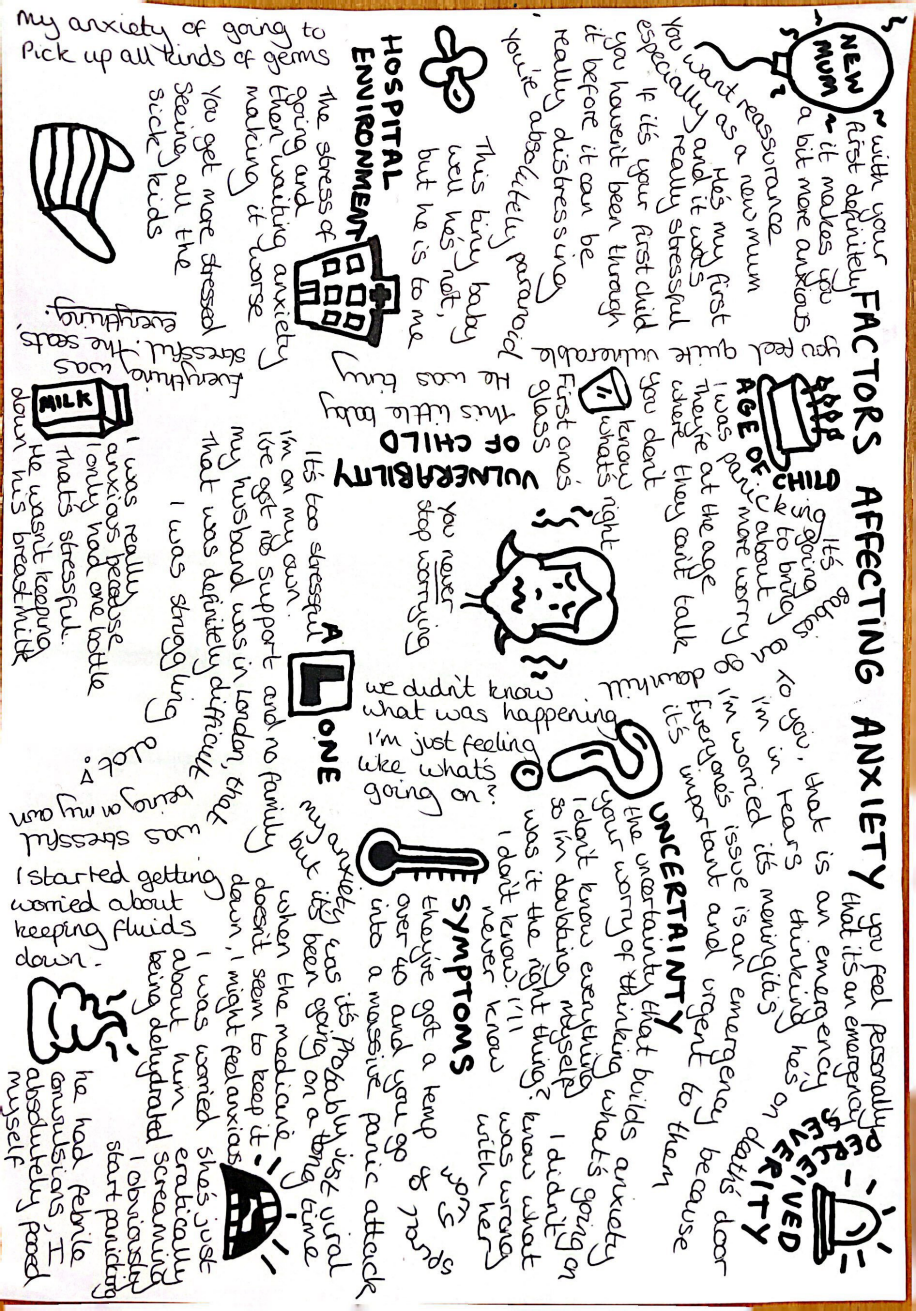
Familiarisation doodle of factors affecting parental anxiety.

Data were analysed using reflexive thematic analysis^20^ led by CF and supported by BC using NVivo V.12Plus (Lumivero, Denver). Transcription of audio recordings facilitated initial familiarisation. Inductive coding, development and refinement of themes occurred through data immersion. The coding structure from parent interviews informed the coding of doctor interviews. A subset of anonymised transcripts was shared with PM to gain parental insight and interpretations. Doodles facilitated critical engagement and sense-making of emerging patterns (figure 1).

Codes were used to ensure anonymity of participants; Mo, mother; Fa, father; C, consultant; JD, resident junior doctor, M, male; F, female (followed by participant number).

Potential biases and assumptions made by the research team were conscientiously considered; the diversity of disciplinary/academic background (mathematicians, paediatricians, children’s nurse and parent) aimed to ensure integrity. The lead researcher’s (CF) reflexive notes contributed to discussions supporting interpretative decision-making. Representativeness and diversity of experience are ensured by selecting quotations from across the sample.^21^

## Results

### Participant characteristics

20 people participated: 15 parents (12 mothers, 3 fathers; 5 first-time parents; 14 from Northwest and 1 London); 5 doctors (4 consultants, 1 resident junior doctor, located in North West EDs; 4 from a paediatric tertiary hospital and 1 participant worked in a mixed ED) (see online supplemental file for participant characteristics). Recruitment opened in June 2022 and closed in January 2023 after the minimum desired number of participants were recruited. Despite the original intention to recruit up to 30 participants (15 parents/carers and 15 HCPs), recruitment—particularly of HCPs—was extremely slow, despite readvertising on social media platforms and expanding the target location. Eventually, recruitment closed to allow sufficient time to analyse and write up the research.

**Figure 2:**
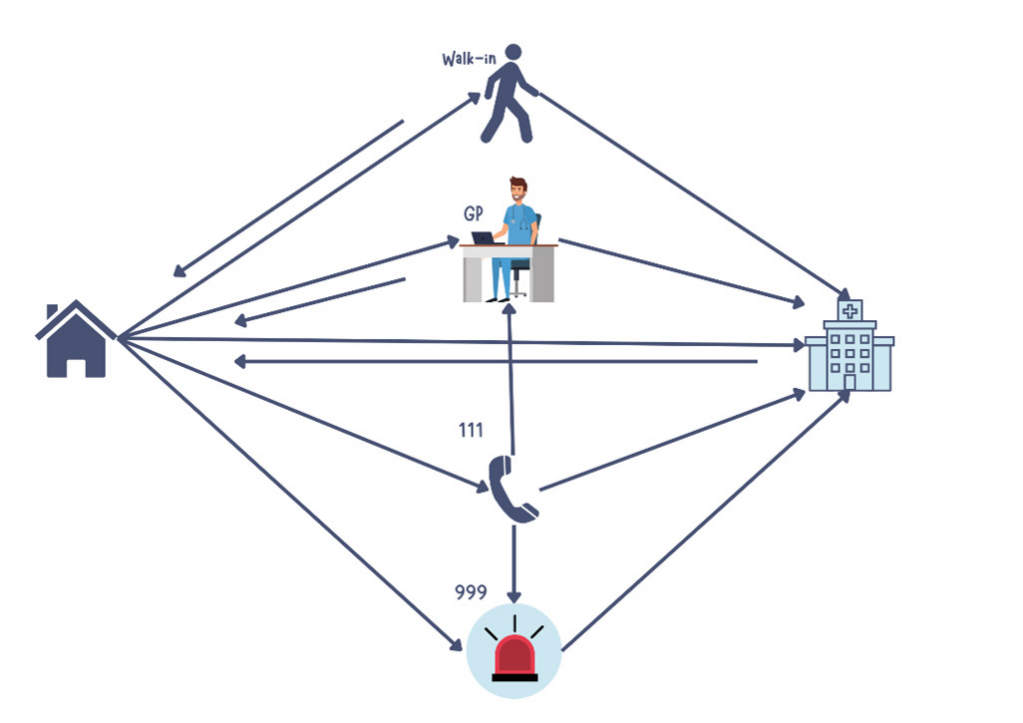
Typical pathways experienced in navigating child’s journey to ED. ED, emergency department.

Findings reveal the services used and pathways (walk-in, general practitioner, NHS111 (online medical advice), 999) typically taken by parents (see figure 2).

### Factors influencing unscheduled care

**Figure 3:**
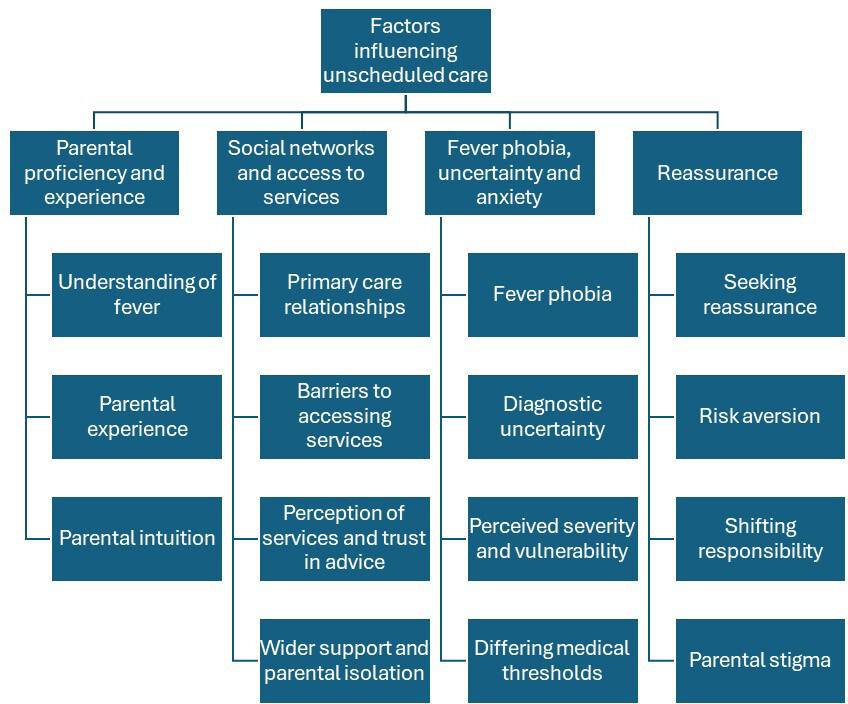
Overarching theme and main and subthemes.

One overarching theme and four main themes were generated (figure 3). All of these reflect the complex interplay between factors at the individual and wider structural level influencing parental decision-making to seek unscheduled emergency care for their febrile child.

### Theme 1: parental proficiency and experience

Perceived proficiency in managing a febrile child was a key indicator of how comfortable parents felt when taking care of their child, and what healthcare advice they sought.

#### Understanding of fever

Prior to any additional symptoms, parents displayed a good understanding of asymptomatic fever, typically seeing it as ‘the body just fighting something’ (Mo,7) and understanding a ‘temperature (of) 38 doesn’t really tell you much’ (Fa,8). Parents seemed comfortable with fever management and administering antipyretics, ‘dosing them up on Calpol’ (Fa,15), providing they perceived the fever as non-threatening. Differing levels of knowledge and practice surrounded the use of antipyretics; some parents did not indicate alternating ibuprofen and paracetamol, and others withheld antipyretics.

Despite parents sharing robust insight about the difference between a high temperature and a fever that needed attention, doctors’ responses suggested ‘not all (parents) know that fever is actually a temperature of 38 or more‘(JD,M,2) and were intolerant of fever that ‘doesn't come down or stay down with Calpol’ (C,F,5).

Apart from this, asymptomatic fever did not heighten concern or trigger parents’ decision to seek medical care but prolonged persistent fever that ‘doesn’t come down after an hour or two’ (Mo,5) or ‘a mixture of things’ (Mo1) triggered help-seeking.

#### Parental experience

Previous fever experience and knowing ‘what to look for’ (Mo,6) made parents feel more proficient. First-time parents tended to not know ‘what was right and wrong’ (Mo,12). Doctors highlighted the importance of parents being able to know ‘what is a normal, hot, grumpy, but okay kid’ (C,F,1) and took parental experience into account when assessing a febrile child. Parents’ perceptions of their child’s vulnerability seemed to reflect experience, ‘first one’s glass, second one’s rubber’ (Fa,9), which was also acknowledged by doctors.

#### Parental intuition

Most parents thought that they would intuitively know when their child ‘weren’t right’ (Mo,1) and when to seek urgent care, meaning some confidently bypassed primary care but some started ‘doubting myself’ (Mo,10). Parents wanted doctors to acknowledge their intuition and holistic understanding of their child. Parents’ ‘biological urge to make sure (their) child is well’ (Mo,7) triggered their need to advocate for their febrile child, this increased emotional burden on the parent.

Typically, parental intuition was given little weight in doctors’ clinical decision-making although they described the benefits of listening to parents. Doctors described parental expectations for blood tests, as a ‘proxy for saying, nobody’s listening to me’ (C,F,5).

### Theme 2: social networks and access to services

Access and availability of social and healthcare support were found to impact parental management of fever.

#### Primary care relationships

Most parents described an impersonal negative detached relationship with their GP that lacked continuity as they were ‘just numbers to be gotten through’ (Mo,3) since they ‘never see the same person’ (Mo,3).

#### Barriers to accessing care

Barriers to care included lack of advice, continuity of care, opening times, convenience of services and primary care relationships. Unavailability of timely GP appointments and inability to contact GPs seemed designed to increase frustration and ‘put you off going’ (Mo,3). Seeking unscheduled care without a referral resulted from practices ‘(not being) open’ (Mo,6) or wanting to avoid waiting. Doctors acknowledged bypassing other services increased ED visits and that being unable to access primary care means ED is the ‘first port of call’ (C,F,5) as you ‘can just turn up’ (C,F,4).

#### Perception of health services and trust in advice

Some parents believe paediatric health services offer a higher level of expertise (better access to diagnostic tests, equipment and higher quality care). Non-paediatric settings were perceived as being unable to ‘navigate that grey zone’ (Fa,9). Doctors acknowledged parent preferences but noted misconceptions about levels of expertise.

#### Wider community support and parental isolation

Family and wider social support ‘reinforced’ (Mo,3) appropriate parental decision-making whereas a lack of family/friends could be ‘a big stress’ (Mo2). Doctors identified most ED attendees as ‘families who are relatively socially isolated’ (C,F,1), noting the usefulness of social circles for parental decision-making.

### Theme 3: fever phobia, uncertainty and anxiety

Feelings of uncertainty, anxiety and fever phobia were the predominant emotions felt by parents when treating their febrile child, as well as during their child’s hospitalisation.

#### Fever phobia

Despite acknowledging fever was not the primary element of their child’s illness, uncertainty and a degree of fever phobia were evident among parents who were concerned it could indicate ‘something worse’ (Fa,8). ‘Scaremongering’ news about children dying heightened vigilance and robust advocacy for their child.

Doctors described a fever paranoia and concern it could indicate a ‘serious infection’ (C,F,3) as a contributing driver for ED attendance. Doctors noted that some colleagues had fever paranoia.

#### Diagnostic uncertainty

Parents were often confused about ‘what does viral mean’ (Mo,12) and questioned whether it is a ‘proper diagnosis’ (Fa,9). Parents wanted a clear diagnosis to improve their own understanding; doctors noted ‘if you just say it’s a viral illness, [parents] … absolutely hate it’ (C,F,5). Non-specific viral diagnoses potentially resulted from ‘(new guidelines that) de-emphasised finding a source’ (C,F,1).

#### Perceived severity and vulnerability

Perception of the severity and/or vulnerability of their child often initiated parental health-seeking behaviour. Parents talked of babies and younger children having increased vulnerability and fragility as they ‘can go downhill quickly’ (Mo,5) and ‘can’t talk’ (Mo,6). Febrile convulsions were ‘absolutely terrifying’ (Mo,7). Doctors acknowledged convulsions as a cause for parental concern but did not share this concern.

#### Differing medical thresholds and advice

Fragmented healthcare services, differing medical thresholds for fever and incomplete or inconsistent information from professionals across different services generated feelings of frustration, anxiety and confusion. Doctors acknowledged that conflicting advice ‘loses a lot of trust in us’ (C,F,3); this was particularly evident in parents who noted a lack of continuity of care and positive relationships with primary care services.

### Theme 4: reassurance

Parents health-seeking behaviours for their child are influenced by their need for reassurance.

#### Seeking reassurance

Parents wanted ‘peace of mind’ (M01), reassurance it was ‘nothing serious’ (Fa,9) and they were ‘doing the right thing’ (Mo3) although reassurance in an ED was described as ‘a waste of resources’ (Mo,3). Doctors noted parents sought ‘professional validation’ (C,F,3) and acknowledged reassurance could ‘re-empower parents’ (C,F,1).

#### Risk aversion

Deciding to ‘go and be on the safe side’ (Mo,3) was driven by risk aversion and uncertainty perhaps reflecting ambiguous advice about fever thresholds. Risk aversion and distrust of professional advice was highest in parents with previous negative experiences of health services. Doctors linked the ‘better safe than sorry’ (C,F,1) approach with meningococcal septicaemia campaigns which ‘over skewed education to just get it checked out, just in case’ (C,F,1). Doctors’ uncertainty arose from remote examinations and fear of disciplinary action for incorrect clinical assessment.

#### Shifting responsibility

Parents noted that ‘no one (GP/ED) wants to deal with you’ (Mo,3) but engaging with a trusted HCP created ‘huge relief’ (Mo,13) and a shift in responsibility. Expertise within paediatric ED was perceived as a trigger for GP referrals.

#### Parental stigma

Stigma associated with being perceived as a ‘fussy mum’ (Mo,6) ‘(feeling) like we shouldn’t really be there’ (M05) influenced parents’ decision-making, especially on return visits when they worried they would be asked ‘Why are you here again?’ (Mo,12). Several parents described embarrassment when their child ‘really perked up’ (Fa,15) in ED (see online supplemental file for additional supportive quotes).

## Discussion

These findings form the first component of a qualitative study to investigate parental and doctor perceptions of reasons for ED presentation for paediatric fever in England. We reflect the findings that pathways to the ED were complex and multifaceted.^15^ Parents wanted to be empowered to take informed responsibility for their child’s care^22^ and to do the ‘right’ thing, suggesting a need for better education on the warning signs for fever, and where parents should first seek help.^23^

As reported elsewhere, our parents experienced anxiety, fear and uncertainty.^24 25^ Confidence was weaker among first-time parents^26^ and those who had received inconsistent information from HCPs.^27^ We mirror findings that anxiety, fever phobia and risk aversion heightened vigilance in parents’ health-seeking behaviours, driving ED attendance.^28^ Accompanying symptoms and the possible complications heightened parental fear.^29 30^ Febrile seizures can be frightening for parents, despite usually being harmless. Providing clear information to parents including the risk of recurrence and how to manage them and providing emotional support could reduce inappropriate use of healthcare and associated costs.^31 32^ ED doctors perceived fever phobia and risk aversion existed among parents and HCPs (in both community and non-community settings), noting this encouraged emergency care use.^5 13^

Despite previous research indicating convenience and ease of access as facilitators for paediatric ED use,^33 34^ ED attendance was a last resort for our parents despite the difficulties they experienced navigating the healthcare system (eg, barriers to accessing GP, lack of familiarity or trust with HCPs), as reported elsewhere.^35^ Successful healthcare experiences and positive long-term outcomes are driven by good communication and relationship building with HCPs^24 36^; these factors should be a priority.

Parental intuition encouraged confidence in seeking appropriate care^37^ including concerned ‘gut feelings’ driving ED attendance.^28^ We support the importance of validating parents’ concerns by listening to and involving them in decision-making throughout their child’s fever pathway.^24^ Diagnostic uncertainty influenced parental understanding of fever; a ‘just viral’ or unspecific diagnosis made parents feel their concerns had been trivialised, as reported elsewhere.^29 38^

HCPs need to provide clear consistent fever-related information (eg, the warning signs which would justify emergency attendance). Eliminating conflicting information would support parental decision-making, encourage trust in HCPs^24^ and prevent selective use of emergency services.

Limitations relate to the bias of convenience sampling, sample size and generalisability of results. Information on socioeconomic status (SES) was not collected from participants. However, recruitment advertisements targeted geographical locations with higher deprivation, such as Liverpool city centre so the sample potentially indirectly reflects deprivation. Previous research notes ethnic differences in parental perceptions and management of paediatric fever.^39^ Investigation of this was not possible in our small sample, and this should be considered in future research. Finally, due to the nature of the methods used within this study, there is a possibility of social desirability bias, as respondents may have given answers they believed presented themselves in sociable acceptable terms. This was mitigated by sensitive engagement with participants. The sample included few fathers or male doctors and only one resident doctor. Three of the five doctors worked within the ED of the same paediatric tertiary hospital, which is not typical of ED settings in England. Further heterogeneity within the sample of doctor participants could have provided richer data. However, the sample of parents was strong and heterogeneous, including single and married/partnered parents, young and older parents, first-time parents and those with more than one child and parents with different experience in managing fever and with different healthcare journeys.

Most participants were located in the North West of England. Further qualitative work in other populations across England would improve the generalisability of results and help to investigate parental reasons for ED attendance for children with febrile illness. For some of the participants, there was a time gap between admission and interview which may impact recall of parent perceptions and decision-making. However, this evidence points to the emotional significance of ED visits,^40^ which may make them more memorable over time. Additionally, using probing questions to cue specific aspects of their experience helped to stimulate responses.

This study widens the evidence base of why parents attend ED for fever in England and their perceptions of other health services. Parents face challenges when seeking care and perceive ED as a last resort. Clear and consistent information is needed to support parental decision-making and potentially reduce the burden on emergency care in England. A novel approach of emotional journey mapping was also used with these participants, to provide further insight into the pathways taken for parents. We hope to report these findings in a later publication.

ED clinicians and parents face the challenge of discerning serious cases from the many minor illnesses that present, balancing the need for caution in identifying potentially severe conditions with the pressures of maintaining efficient service delivery under high patient volumes. Interventions supporting parental decision-making and fever management could help to alleviate these challenges, and potentially reduce the demand for emergency care.^41^

Stronger support in the community for basic home management of acute illness could have a significant influence in strengthening parental confidence and management of paediatric illnesses and encourage appropriate health-seeking behaviour. Building and maintaining trust and positive relationships between parents and HCPs should be a priority.

Further research should investigate the effect of low SES in the management and decision-making for febrile children as well as investigating specific communities and populations such as ethnic minority groups and marginalised or stigmatised groups(such as asylum seekers, non-English-speaking groups), to help uncover possible intersectional factors at play in parents’ decision-making throughout the parents’ journey to ED.

## supplementary material

10.1136/bmjpo-2024-003039 online supplemental file 1

## Data Availability

All data relevant to the study are included in the article or uploaded as supplementary information.
