# Supplementary material for: Coming in hot: a qualitative investigation into perceptions of parents and doctors of reasons for the presentation of children with fever to the emergency department in England
Source: BMJ Paediatr Open. 2024 Dec 24;8(1):e003039. doi: 10.1136/bmjpo-2024-003039 (PMC11683883; doi:10.1136/bmjpo-2024-003039)
Supplement: online supplemental file 1 [file bmjpo-8-1-s001.pdf]

SUPPLEMENTATY FILE

Parent characteristics

Participant	Participant's gender	Location	First time parent	Number of child experiences discussed
Mo,1	Female	Liverpool	No	1
Mo,2	Female	Liverpool	No	2
Mo,3	Female	Greater Manchester	Yes	1
Mo,4	Female	Cheshire	Yes	1
Mo,5	Female	Liverpool	No	2
Mo,6	Female	Liverpool	No	2
Mo,7	Female	Lancashire	Yes	1
Fa,8	Male	Liverpool	No	1
Fa,9	Male	Wirral	No	1
Mo,10	Female	Liverpool	No	1
Mo,11	Female	Liverpool	No	2
Mo,12	Female	Lancashire	Yes	2
Mo,13	Female	London	Yes	1
Mo,14	Female	Liverpool	No	1
Fa,15	Male	Liverpool	No	1

Mo= mother, Fa= father

Doctor characteristics

Participant	Level of profession	Gender	Location
C,F,1	Consultant	Female	Lancashire
JD,M,2	Junior Doctor	Male	Liverpool
C,F,3	Consultant	Female	Liverpool
C,F,4	Consultant	Female	Liverpool
C,F,5	Consultant	Female	Lancashire

C=consultant, JD= junior doctor, F=female, M=male

Supporting quotes for each theme

Theme 1: Parental proficiency and experience
**Understanding of fever**<br>*Parents' perspectives*<br>• "A temperature of like 38, 39 it doesn't really give me much cause for concern. It's when it's going over 40 that I'm a little bit more concerned because I'm thinking well her body is obviously struggling a little bit more there" (Mo,7).<br>"With the mixture of the things that just like felt like more needed to be looked at" (Mo,1).<br>*Doctors' perspectives*<br>• "Not all of them know that fever is actually a temperature of 38 or more. And when we ask them, they say 37.3, 37.5, which is low grade pyrexia, but it's not actually fever" (JD,M,2)<br>• "It [ED presentation] can be because of things that we would worry about. So, like the child is lethargic, or they're not eating and drinking, and they're not passing urine, things like that" (C,F,3).
**Parental experience**<br>*Parents' perspectives*<br>• "You build up a knowledge base of illnesses and what to look for" (Mo,6).<br>• "I'd never experienced it before...I didn't know what was right and wrong" (Mo,12).<br>• "That old analogy, first one's glass, second one's rubber…You're absolutely paranoid with the first child that the slightest little knock might kill him straight off…They're not actually as fragile as you would lead yourself to believe when they're your first one" (Fa,9).<br>*Doctors' perspectives*<br>"If people don't live, close knit with their kind of nieces and nephews and other people's small children, they don't experience small children nearly as much till they suddenly have their own and they're responsible for them. there're less just battleground miles on the clock of what kids look like. And what is a normal, hot, grumpy, but okay, kid?" (C,F,1).<br>"If it's a parent that's had three more children, they've been through many infections, and they are concerned at this time that their child looks unwell, that's more serious" (JD,M,2).
**Parents' intuition**<br>*Parents' perspectives*<br>• I am a genuine believer in a lot of cases of mother's intuition is right, that there is something wrong" (Mo,7).<br>• "I know them [child] better than the doctor" (Fa,8).<br>• "Listen to parents, they know their child. You might know like, the physiology of it but we know our children…I know when there's something wrong with her. And I do feel like had we'd been listened to properly it would never have escalated as much as it did" (Mo,7).<br>*Doctors' perspectives*<br>• "if you get the listening to concerns right, that's probably quite therapeutic parents….. [it] down regulates the expectations of drugs" (C,F,1).

- “a biological urge to make sure your child is well” (Mo,7).

Theme 2: Social networks and access to services

Primary care relationships

Parents’ perspectives

- “awful framing of people as numbers to be seen. That means that people aren't people anymore. They're just numbers to be gotten through as quickly as possible and issues to be resolved as quickly as possible through whatever route is not currently over demanded for” (Mo,3).

Barriers to accessing care

Parents’ perspectives

- “access to that [GP] advice can be a bit tricky sometimes” (Fa,8).
- “I thought that this wasn't a walk-in centre type thing to do. Because they would’ve just referred us to [paediatric hospital], and we would've just been sat in the waiting room, and we wanted to bypass that” (Fa,8).

Doctors’ perspectives

- “the urgency with ease of being seen” (C,F,4).

Perception of health services and trust in advice

Parents’ perspectives

- “I always tend to go to the walk-in centre… because they're so knowledgeable there” (Mo,6)
- “I was more happy that they did actually give me antibiotics…perhaps maybe if I'd have taken him around one of his doctors, they may not have been so willing” (Mo,6).
- “I wasn't happy with how the other A&E had dealt with it. So I thought, no, you know what, we'll go back to [second paediatric hospital name]” (Mo,6).

Doctors’ perspectives

“there's also lack of understanding… you might see a paediatrician who's in the first year of their training” (C,F,4).

Wider community support and parental isolation

Parents’ perspectives

- “My mum came… having that conversation, kind of reinforced, I am making the right decision” (Mo,3).
- “So not having anyone around to just go 'Am I being reasonable or unreasonable? What's the right approach here?' That was definitely difficult being on my own…Definitely having someone around would have been easier. Just to bounce the ideas around rather than making the decisions all on my own easier” (Mo,3).

Doctors’ perspectives

- “the lack of health visitors over that time was massive, much more than lack of doctors...people really, really struggled” (C,F,5).

Theme 3: Uncertainty and anxiety

Fever phobia

Parents’ perspectives

- “[fever] could be because of something very little… and the other it can also be the sign of something extremely serious” (Mo,7)

- “that one child who, who had a bacterial thing when you said it was viral, and then was sick and died or something” (Fa,8).
- “What does viral mean? … there's no explanation on that either…It's just viral. It's hard to get your head around it.” (Mo,12)

Doctors’ perspectives

- “they're worried that the child has meningitis or appendicitis or you know, another serious infection that's going on?” (C,F,3).
- “probably frightened parents about fever…They're really scared about the fever” (C,F,1).
- “We as a profession have, there's a little bit more of a move to getting rid of our fever paranoia as well” (C,F,1).

Diagnostic uncertainty

Parents’ perspectives

- “What does viral mean? … there's no explanation on that either…It's just viral. It's hard to get your head around it.” (Mo,12)
- “I want to know the cause [of fever]. I think that's more my issue … I want to know the cause, to understand” (Mo,5).

Doctors’ perspectives

- “being unable to find the source of the fever was kind of a bad thing with capital B, and T... [new guidelines] deemphasized finding a source” (C,F,1).
- “Some other people will diagnose them with a viral illness, even if there's no signs of virus, because that's a diagnosis. And I think parents then feel a bit better because if they've got a diagnosis” (C,F,3).
- “'Stop telling me it's a viral illness'. And you kind of say, well, 'actually, this is just the immune system working. You know, I think they're okay, this is just a virus” (C,F,4).

Perceived severity and vulnerability

Parents’ perspectives

- “I'd never heard like, never seen anything like that [convulsion] before. So it was really scary” (Mo,12).
- “so when she was a new born baby, Yeah, [paranoia] definitely did come in because obviously anything when they're that little is just like ‘Oh my god’” (Mo,7).
- “he's this tiny little baby. Well, he's not, he's two but he is [a baby] to me” (Mo,11).

Doctors’ perspectives

- “one very common thing that parents are concerned about” (JD,M,2)
- “babies are the ones that you kind of worry about a little bit more, and particularly the neonates” (C,F,4).

Differing medical thresholds and advice

Parents’ perspectives

- “the GP doesn't want to deal with it, because you've reached a [temperature] threshold that says no [to being treated by the GP]” (Mo,3).
- “we were referred [to ED] from the GP. And it was like, in our minds, it was an urgent referral, because we were really worried about her. And they like really layered it on at the GP of how concerned they were…we assumed we'd go to the GP and home again for dinner” (Mo,5).

Doctors’ perspectives

- “they thought that they had to go to A&E, because that's what they were told. And it's quite frustrating” (C,F,3).

Theme 4: Reassurance

Seeking reassurance

Parents’ perspectives

- “I don't care if you think it's nothing. Unless you can show me It's nothing. I'm not gonna be okay'” (Mo,10).

Doctors’ perspectives

- “Some would come just because they want reassurance and they want to know that their child's okay” (C,F,3).
- “to look them in the eye and tell them that their child is going to be okay…and guarantee it...you can’t really do that” (C,F,4).

Risk aversion

Parents’ perspectives

- Q6. “just go and be on the safe side” (Mo,3).

Doctors’ perspectives

- Q7. “lack of seeing the bigger picture” (C,F,1).
- Q8. “we've over skewed the education to just get it checked out just in case” (C,F,1).
- Q9. “You know when we went a few days? Well, we didn't really mean it would definitely be gone by day five” (C,F,5).
- Q11. “If you've not seen them, you have to be much more cautious…And they [GP] have a different I think to some extent they have a different level of risk tolerance” (C,F,5).

Shifting responsibility

Parents’ perspectives

- “it baffles me of no one really wants to deal with you. They don't want you in A&E, but they don't want you at the GP surgery. So what are you supposed to do?” (Mo,3).

Doctors’ perspectives

- “[GPs] don’t want to take that final decision” (C,F,4)
- “[GPs] will always say, in as a caveat to their advice. 'If they don't get better go to [hospital]” (C,F,4).

Parental stigma

Parents’ perspectives

- “am I wasting time” (Mo,3)
- “there's this little you get this little niggle inside you and like if I don't get this checked and something bad happens. I'll never forgive myself. So you kind of that instinct kicks in and you go, it doesn't matter whether you're embarrassed or not. You have to go” (Mo,11).
- He'd really perked up when he got there and sort of playing around when he was little. And I felt like I'm being a bit of a fraud” (Fa,15).

Doctors’ perspectives

- “Sometimes…a lot of times, … they'll come into ED, they'll get some ibuprofen or paracetamol at triage, and then they'll be running around in the waiting room by the time I called them through. And the parents were like, 'Honestly, they weren't like that before. They just were honestly literally

sick before'. And you've got to kind of reassure them that 'No, that's okay. We know that sometimes [they] look better by the time they come in here" (C,F,3).

- "that they're not judged…… [and that you] "re-empower the parents and…. verbalizing that you trust their judgment about their kid" (C,F,1).
- A lot of the times it is reassuring them that they've not done the wrong thing by coming. Because I think, like you said, they thought long and hard about it before they came. And they felt like that was the right thing to do" (C,F,3).

Parent advert

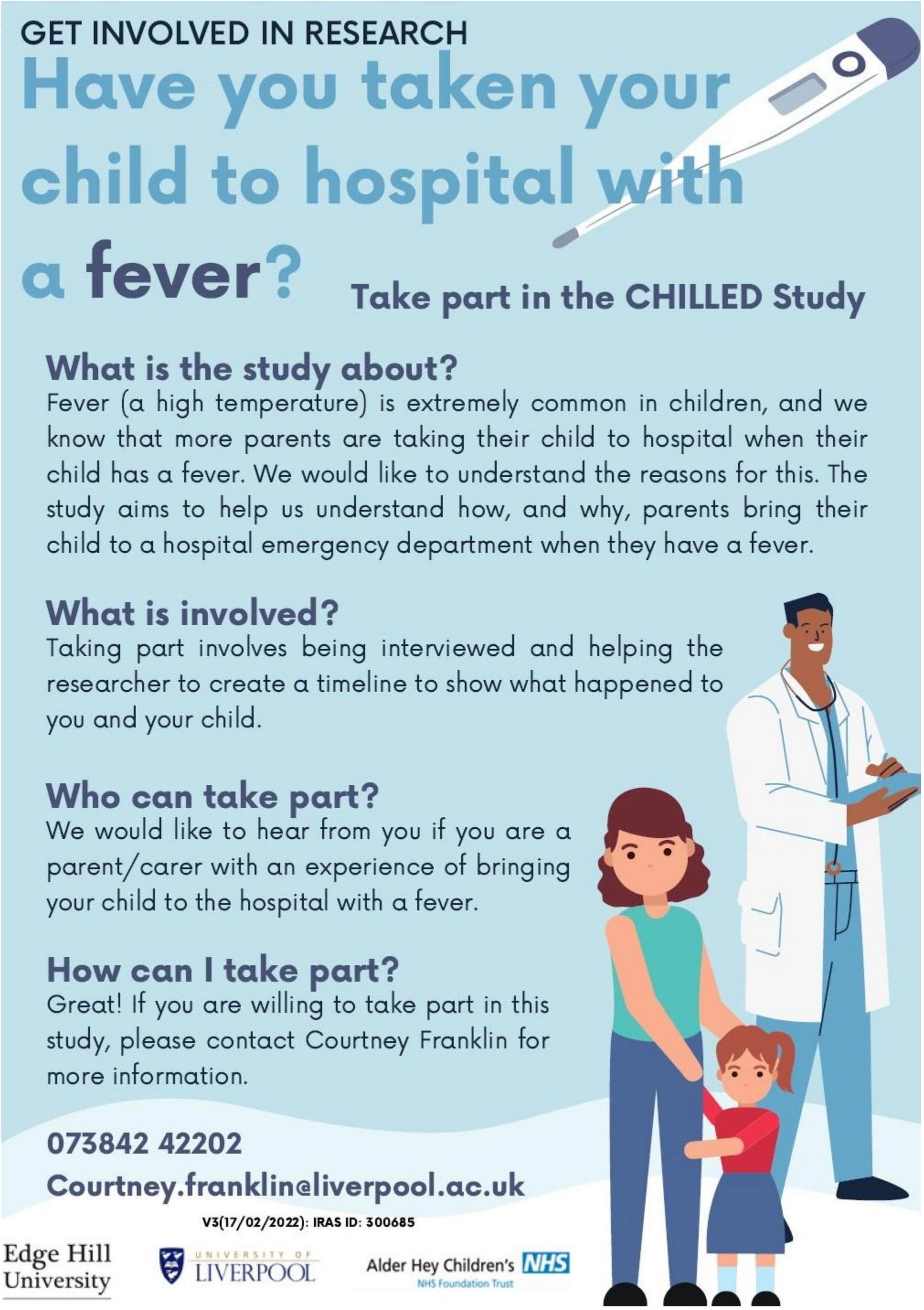

Parent consent form

FUNDED BY NIHR | National Institute for Health and Care Research   Edge Hill University   UNIVERSITY OF LIVERPOOL   Alder Hey Children's NHS Foundation Trust

CHildhood Inequalities in Liverpool in febriLe attendances to the Emergency Department

Consent Form: Parent/carer

Study Team: Courtney Franklin, Prof David Taylor-Robinson, Prof Enitan Carrol and Prof Bernie Carter

	**Please initial box**
I confirm that I have read and understand the Information Sheet (version number, date) for the above study and have had the opportunity to ask questions.	
I understand that my participation is voluntary and that I am free to withdraw up to 7 days after I have been interviewed without giving any reason.	
I understand that my interview will be audio recorded and will form part of the data collection for this study.	
I understand any timeline of the reason for my attendance at the emergency department that I help create during my interview will form part of the data collection for this study.	
I understand that some of the things that I say (in the interview) may be quoted in publications and I understand that these quotations will be anonymised and that no personal information will be used in the study report or publications.	
I understand that the image of my timeline may be included in publication. I understand that this be anonymised. None of my personal information will be used in the study report or publications.	
I understand that my consent form will be stored electronically, and the original paper copies will be destroyed in an appropriate manner. Further information can be found in the information sheet.	
I understand that anything that I say during the interview which raises safeguarding concerns may need to be addressed by the researchers and that may include breaking confidentiality to share that information for the safety of my child.	
I understand that the data collected during the study may be looked at by individuals from regulatory authorities or from the NHS Trust or from the University of Liverpool. I give permission for these individuals to have access to the records.	
If I would like to receive details of the results of the study including any research produced from it, I can access the CHILLED Study Facebook page.	
I agree to take part in the above study.	

Name of Parent/Carer……………………………Date……………Signature………………..

Name of Researcher…………………………Date………………Signature……………………….

IRAS ID: 300685   CONSENT FORM PARENT/CARER 12_01_2022 V1.0

Parent pre-interview information sheet

CHildhood Inequalities in Liverpool in febriLe attendances to the Emergency Department

(CHILLED Study)

Information sheet for parents/carers

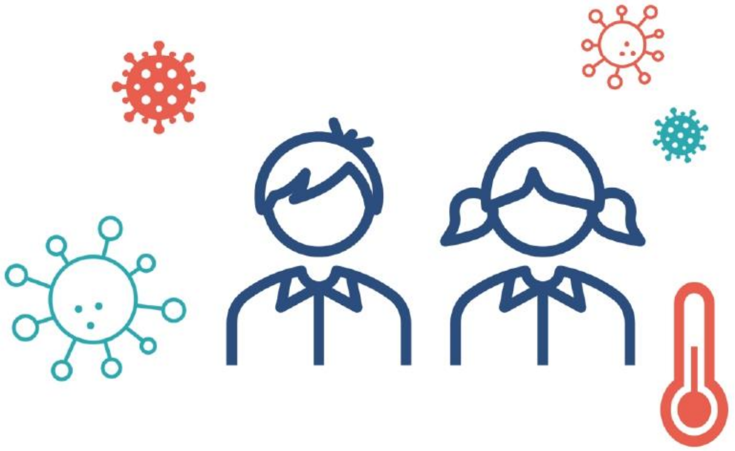

Study Team: Courtney Franklin, Prof David Taylor-Robinson, Prof Enitan Carrol and Prof Bernie Carter

Introduction

We are asking if you would like to take part in a study. Before you decide if you want to take part you need to know why we are doing the study and what will happen. This leaflet tells you all about the study.

Fever (a high temperature) is extremely common in children, and we know that more parents are taking their child to hospital when their child has a fever. We would like to understand the reasons for this.

What is the study about?

The study aims to help us understand how, and why, parents bring their child to a hospital emergency department when they have a fever.

Who is doing the study?

The people who are doing the study are called Courtney Franklin and Prof David Taylor-Robinson from the University of Liverpool, Prof Enitan Carrol from Alder Hey Hospital and University of Liverpool, and Prof Bernie Carter from Edge Hill University. One of the study team will talk to you and about your possible involvement in the study.

Why have I been asked to take part?

We are asking you to take part because you have experience of taking your child to hospital when they had a fever.

Do I have to take part?

No! It's up to you whether you take part in the study. If you do take part, you can change your mind up to 7 days after you have been interviewed, without telling us why and no one will mind.

What will happen to me if I decide to take part?

Taking part involves being interviewed and helping the researcher to create a timeline (a drawing that shows what happened).

The interview

We would like you to take your time to get your experience across. The interview could take up to 60 minutes but if you don't have so much time, we'd still love to talk to you for 10 or 15 minutes. We would like you to take your time to get your experience across. We would prefer to conduct your interview face-to-face, at an arranged time, in a convenient place (e.g at a children's community centre). However, if you prefer, we will carry out these interviews virtually, via Zoom, What's App (or similar) or by phone.

- We will ask about who you asked for advice about your child's fever.

- We will ask about why you took your child to an emergency department and what your experience was like.
- We will ask about the treatment your child may have received (e.g. antibiotics) and what you thought about it.
- We will ask about what things were helpful and what things weren't so helpful.

The timeline

The timeline will happen as part of the interview. It will help us to be clear about what happened and when it happened, from the time your child started to get poorly to your child being in the emergency department. It will also help us see how you were feeling at the time. The researcher can help create this. The picture below gives an example of what a timeline might look like.

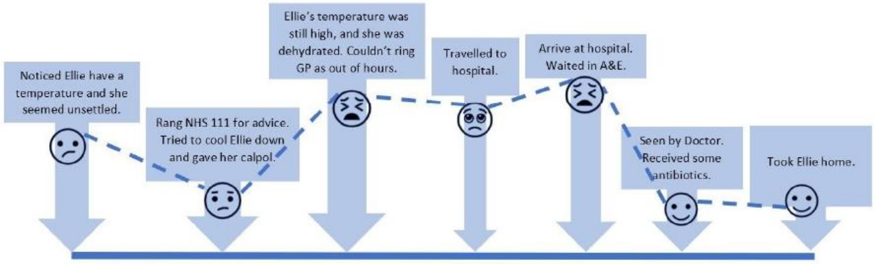

We would like to audio-record interview so we can remember the important things you have told us. If you do not want to be audio-recorded, then you can tell us and we will just make notes of what you say instead. After the interview, the audio-recordings will be typed up and all names and identifying information will be removed. The audio-recording will then be destroyed. Any identifying information on your timeline will be removed.

How do I give consent to take part?

If you are willing to take part in this study, then you can contact Courtney Franklin (contact details provided below). We will ask you to sign a consent form before the interview.

If you change your mind about taking part this is fine, you can change your mind at any time before the interview or up to 7 days after the interview.

What are the possible disadvantages and risks of taking part?

We do not think that there are any disadvantages or risks for you taking part in this study. We will take great care of the information that you tell us.

What are the possible benefits of taking part?

We hope that you will enjoy taking part in this study. The information that you share with us could help make things better for parents/carers when they are deciding what help to seek if their child has a fever. It could also help us to improve future experiences for parents/carers and their child whilst in hospital for a fever. To show our appreciation for your contribution to the study, you will have a chance to win a £25 voucher. If you win, you will be contacted after all interviews are complete.

What happens when the study stops?

Once you have taken part in the interview you will not need to do anything else. We plan to have finished the study and written all the reports by September 2023. If you would like, we will send you a short summary of the study when it is finished, just let us know.

Will my taking part in this study be kept confidential?

The University of Liverpool is the sponsor for this study based in the United Kingdom. We will be using information from you in order to undertake this study and will act as the data controller for this study. This means that we are responsible for looking after your information and using it properly. The university will only keep your personal contact details until the interview is completed. This is in case we need to contact you. We will keep your name and contact details confidential and will not pass this information to unrelated parties.

All the information that is collected during the study about you and your child will be kept strictly confidential and your name and your child's name and other personal details will not be shared in any final reports, presentations, articles or other outputs we create. If you were to share with us any information about poor practice or illegal activity or we are worried that someone is being harmed, then we would have to share this information with professionals outside the study team. We would talk to you about this before we took any action.

The research student and the supervisory team will be able to access the data you provide (timeline and audio-recording of your interview) using secure University systems. As soon as audio-files have been transferred to the secure server they will be deleted from the recording device. The audio-files will be deleted from the secure server once the anonymised transcripts have been checked for accuracy and omissions. Your timeline will be anonymised. Your rights to access or remove your information are limited, as we need to manage your information in specific ways in order for the research to be reliable and accurate.

If you withdraw from the study, we will keep the information about you that we have already obtained, unless you tell us within 7 days of the interview that you don't want us to use it. To safeguard your rights, we will use the minimum personally-identifiable information possible.

Your consent form will be stored electronically and the original paper copies will be destroyed in an appropriate manner. Data and all appropriate documentation will be stored for a minimum of 10 years. After this, it will be destroyed securely, in line with the university protocols.

What will happen to the results of the study?

The results of the study will be shared across the NHS and will inform parents' views and experiences of managing their child with a fever. We plan to write articles and present the work to help us share our ideas with other professionals. Quotations of things you may say in the interview and an image of your timeline may be included in the publications but these will be anonymous and you and your child will not be identifiable from them.

Who is funding this study?

This study is part of a larger PhD research project. It is jointly funded by NIHR SPHR and NIHR ARC NWC.

Who has reviewed the study?

The study has been reviewed by the research study team, by an internal review at the University of Liverpool, and by the Health Research Authority, Research Ethics Service (committee details and numbers to be added).

Where can I find updates about the study?

This study is part of a wider research project. If you want to keep updated about the study, you can find information at https://arc-nwc.nihr.ac.uk/impact/our-phd-students-research/febrilestudy/

Who can I contact if we want to take part or want more information?

If you want to take part, or you would like us to tell you more about the project and answer your questions, please contact:

- Research Student, Courtney Franklin
  (073842 42202/Courtney.franklin@liverpool.ac.uk)

If you have any concerns about the research and wish to speak to someone independent, please contact sponsor@liverpool.ac.uk.

Thank you very much for your time in reading this information leaflet and for considering taking part in the study.

Parent interview guide

CHildhood Inequalities in Liverpool in febriLe attendances to the Emergency Department

(CHILLED Study)

Interview Guide (Parents)

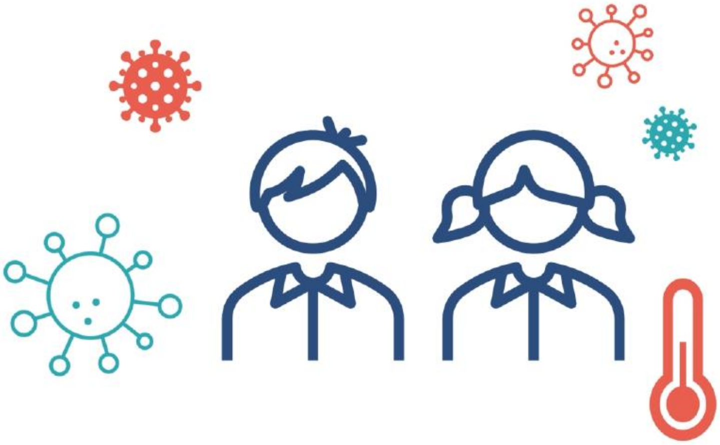

Study Team: Courtney Franklin, Prof David Taylor-Robinson, Prof Enitan Carrol and Prof Bernie Carter

1. Opening Section

The interviewer will summarise the study using the parent/caregiver information sheet, giving the participant an opportunity to ask questions, then gain consent (as per procedure). The interviewer will reassure the participant that there are no right or wrong answers and that they are just interested in their perceptions and experiences.

2. Main Section

The interviewer will invite the parent/caregiver to talk about their perceptions and experiences associated with managing their febrile child. Using a mixture of open, and more directed questions, the researcher will engage the participant in conversation to elicit their perceptions and experiences associated with how and why they sought medical advice at an Emergency Department (ED) for their febrile child. A timeline will be used to help the participant to map their journey and explore who they had consulted for advice, how helpful this was, and what weight they placed on the advice, and how they felt at each part of the journey. The timeline will help unpick issues related to parental concern in terms of fever and management of fever, examining their relationship with their GP/surgery and previous experience of presenting to GP, how seriously they felt their concerns were taken by HCPs.

The following questions and prompts will be used (although they will not necessarily be addressed in this order and the phrasing may change in response to individual contexts).

- Please tell me about [name of child] who developed a fever.
  - How old were they?
- Please tell me about you and your family.
  - Does [name of child] have any brothers or sisters? Are they older or younger?
- What do you know about fever?
- Please tell me about when you first noticed [name of child's] fever.
  - What were they like? Did they seem unwell? Were they experiencing any other symptoms?
- Please tell me about how confident you felt to treat [name of child] at home.
  - What did you know about fever? Have you experienced having a child with a fever before? How do you think is best to treat a child with a fever? Did you have any concerns?
- What options did you feel you had to seek advice?
  - Did your circumstances influence which advice you felt you could seek? Did you contact a GP, pharmacy or use the NHS 111 helpline? If not, why? Do you have a preference of what resource you would use, and why? Was it easy to contact these resources? How accessible were they for you? How do you feel about visiting your GP? Did you feel that your concerns for your child were taken seriously?
- Please tell me about the advice you sought before deciding to take [name of child] to hospital.

    - Why did you seek this advice? Did your circumstances influence your decision making? How useful was any advice you received? How seriously did you take the advice? Did the advice help to settle any concerns you may have had? How did you feel after receiving the advice? Is there anything that could have made this experience easier for you? Did you feel COVID impacted your decision making? How?
- What led you to make the decision to take [name of child] to hospital?
    - E.g. your child's condition deteriorated, you were following medical advice, you weren't happy with the advice you were given from elsewhere, it was the most convenient option.
- Please describe your journey to hospital.
    - How did you get there? How easy/difficult was it to get to hospital? Was there anything that could have made this experience easier? What time of day was it? Did you have any concerns at the time?
- Please tell me about when you arrived at hospital.
    - How long was your wait? What expectations did you have? How were you feeling at the time?
- What happened when [name of child] was then seen?
    - Were you seen by a doctor or nurse? Did you feel this made a difference?
    - Did your child have any tests? How did you feel about this?
    - Was your child given any antibiotics? Did they receive any other medicines or treatments? How did you feel about this?
- How did you feel after you left the hospital?
    - Were you happy/unhappy with the treatment your child received? Did you feel more confident about managing your child at home? Did you feel any concerns you had were acknowledged? Did you receive any follow-up information? How useful was this?
- If you were to experience this again in the future, is there that could have been done differently?
    - Do you feel your circumstances made your experience easier/harder? Do you have a better understanding of managing a child experiencing a fever? Do you feel more confident about managing your child at home? Would you seek advice elsewhere before going to the hospital (e.g contacting your GP)?
- Is there anything that you think could have made the experience easier?
    - E.g clearer information online, better access to out of hours care.
    - Do you feel your circumstances made your experience easier/harder?

3. Closing Section

The interviewer will thank the participant, checking that they are okay and ask if they have any other questions.

Parent post-interview information sheet

National Institute for Health and Care Research (FUNDED BY NIHR) · University of Liverpool · Edge Hill University · Alder Hey Children's NHS Foundation Trust

The CHILLED Study

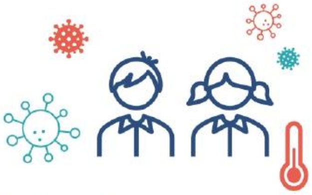

Helpful Information Sheet

Thank you for taking part in the CHILLED study.

Your involvement has been really important.

Are you OK?

We want to be sure that everyone who took part in the study is OK.

We realise that taking part in the study might have caused you to think a bit more about a time that was distressing for you and your child.

What should you do if you are feeling sad or upset?

If taking part in the study has made you feel a bit sad or upset, then we want you to know that you don't have to deal with those feelings by yourself.

Talk to someone you trust about how you're feeling. You might decide to talk to your partner, a friend, someone from your family or someone else you trust, like your GP.

If you don't want to talk to someone you know then you can contact people via support organisations. We have provided details of some support groups you might find helpful.

Some helpful contact details

Action For Children and Mind provide free and confidential advice for parents.
https://parents.actionforchildren.org.uk/
https://www.mind.org.uk/information-support/helplines/

NHS & Alder Hey website provide general advice for fever.
https://www.nhs.uk/conditions/fever-in-children/

https://alderhey.nhs.uk/parents-and-patients-1/fever-high-temperature?q=%2Fparents-and-patients-1%2Ffever-high-temperature

Doctor advert

GET INVOLVED IN RESEARCH

Have you treated a child with a **fever**?

Take part in the CHILLED Study

What is the study about?

The study aims to explore your ideas about reasons why children with a fever are presenting to a hospital ED. We would like to hear about your experiences. Your experience will help us to understand (1) your perceptions about why parents/carers present their febrile child to the ED, and (2) your decision making for the treatment of febrile children.

What is involved?

Taking part involves being interviewed and telling us about what you do, any advice you seek, the decisions you make and your reasons behind performing diagnostic tests, determining a course of action, including whether you prescribe antibiotics.

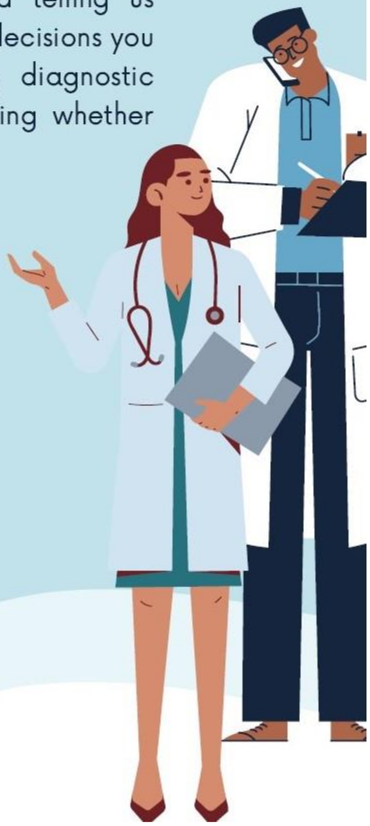

Who can take part?

We are asking you to take part if you are a doctor and have experience treating a child in the ED who has presented with a fever.

How can I take part?

Great! If you are willing to take part in this study, please contact Courtney Franklin for more information.

073842 42202

Courtney.franklin@liverpool.ac.uk

V3 (17/02/2022): IRAS ID: 300685

Edge Hill University | UNIVERSITY OF LIVERPOOL | Alder Hey Children's NHS Foundation Trust

Doctor consent form

Edge Hill University | University of Liverpool | Alder Hey Children's NHS Foundation Trust

CHildhood Inequalities in Liverpool in febriLe attendances to the Emergency Department

Consent Form: Healthcare Professional

Study Team: Courtney Franklin, Prof David Taylor-Robinson, Prof Enitan Carrol and Prof Bernie Carter

	**Please initial box**
I confirm that I have read and understand the Information Sheet (version number, date) for the above study and have had the opportunity to ask questions.	☐
I understand that my participation is voluntary and that I am free to withdraw up to 7 days after I have been interviewed, without giving any reason.	☐
I understand that my interview will be audio recorded and will form part of the data collection for this study.	☐
I understand that some of the things that I say (in the interview) may be quoted in publications/reports and I understand that these quotations will be anonymised and that no personal information will be used in the study report or publications.	☐
I understand that my consent form will be stored electronically, and the original paper copies will be destroyed in an appropriate manner. Further information can be found in the information sheet.	☐
I understand that the data collected during the study may be looked at by individuals from regulatory authorities or from the NHS Trust or from the University of Liverpool. I give permission for these individuals to have access to the records.	☐
If I would like to receive details of the results of the study including any research produced from it, I can access the CHILLED Study Facebook page.	☐
I agree to take part in the above study.	☐

Name of HCP..............................Date................Signature..............................

Name of Researcher..............................Date..................Signature............................

FUNDED BY NIHR | National Institute for Health and Care Research

Doctor pre-interview information sheet

CHildhood Inequalities in Liverpool in febriLe attendances to the Emergency Department

(CHILLED Study)

Information sheet for Health Care Professionals for their own participation in the study

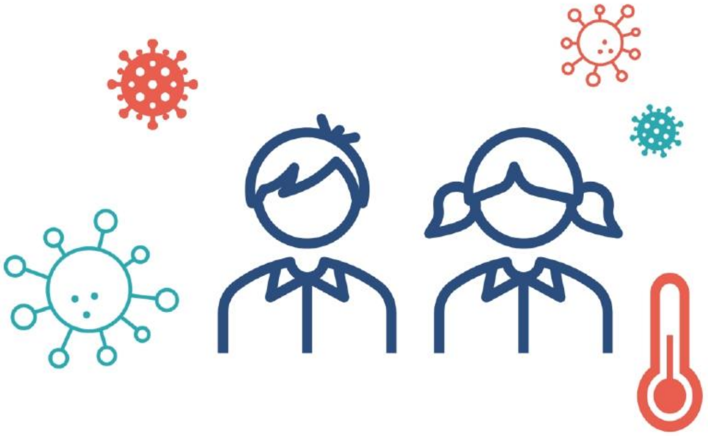

Study Team: Courtney Franklin, Prof David Taylor-Robinson, Prof Enitan Carrol and Prof Bernie Carter

Introduction

We are asking if you would like to take part in a study. Before you decide if you want to take part you need to know why we are doing the study and what will happen. This leaflet will tell you all about the study.

Febrile illness is extremely common in children and increasingly, parents/carers are taking their child to the emergency department (ED) when their child has a fever. Children presenting with febrile illness provide both a diagnostic challenge for healthcare professionals (HCPs) as, although many cases are due to self-limiting viral infections, it can also be a sign of a very serious bacterial infection, of which timely diagnosis and treatment is integral. We would like to understand HCPs perceptions of the reasons for ED presentation, including perceptions about why parents/carers may present their febrile child to the ED, and HCP decisions to perform further diagnostic tests or prescribe antibiotics for children presenting with febrile illness.

What is the study about?

The study aims to explore your ideas about reasons why children with a fever are presenting to a hospital ED. We would like to hear about your experiences of what you do, any advice you seek, the decisions you make and your reasons behind performing diagnostic tests, determining a course of action, including whether you prescribe antibiotics. We hope the study will help us to understand how, and why, parents bring their child to a hospital ED when they have a fever, and how and why decisions are made when performing diagnostic tests or prescribing antibiotics for a child presenting with a fever.

Who is doing the study?

The people who are doing the study are called Courtney Franklin and Prof David Taylor-Robinson from the University of Liverpool, Prof Enitan Carrol from Alder Hey Hospital and University of Liverpool, and Prof Bernie Carter from Edge Hill University. One of the study team will talk to you and about your possible involvement in the study.

Why have I been asked to take part?

We are asking you to take part if you have experience treating a child in the ED who has presented with a fever. Your experience will help us to understand (1) your perceptions about why parents/carers present their febrile child to the ED, and (2) your decision making for the treatment of febrile children.

Do I have to take part?

No! It's up to you whether you take part in the study. If you do take part, you can change your mind up to 7 days after you have been interviewed, without telling us why and no one will mind.

What will happen to me if I decide to take part?

If you take part, you will be interviewed by a member of the study team. It is intended that these interviews will take place face-to-face, at an arranged time and convenient place (e.g. a small meeting room within the hospital). However, if we are unable to conduct interviews face-to-face (e.g. due to social distancing rules), or if you would prefer, these interviews will take place virtually, e.g. via Zoom, or over the phone.

These interviews can then take place at a time most convenient to you. We expect the interview will take about 15 minutes but we can tailor this to suit the time you have available. We would like to talk to you about your previous experience treating febrile children who have presented to the ED and about your thoughts and feelings.

We would like to audio-record your interview so we can remember the important things you have told us. If you do not want to be audio-recorded, then you can tell us and we will just make notes of what you say instead. The audio-recordings will be typed up and all names and identifying information will be removed; the audio-recording will then be destroyed.

How do I give consent for me to take part?

If you are willing to take part in this study, then you can contact Courtney Franklin (contact details provided below). We will ask you to sign a consent form before the interview.

If you change your mind about taking part this is fine, you can at any time before the interview or up to 7 days after the interview.

After the researcher has spoken to you about the study and you have been given the opportunity to give your consent if you wish to take part, we will begin the interview.

What are the possible disadvantages and risks of taking part?

We do not think that there are any disadvantages or risks for you taking part in this study. We will take great care of the information that you tell us.

What are the possible benefits of taking part?

We hope that you will enjoy taking part in this study. The information that you share with us could help make things better for parents/carers when they are deciding what help to seek if their child has a fever and/or future experiences in ED.

What happens when the study stops?

Once you have taken part in the interview you will not need to do anything else. We plan to have finished the study and written all the reports by September 2023.

Will my taking part in this study be kept confidential?

The University of Liverpool is the Sponsor for this study based in the United Kingdom. We will be using information from you in order to undertake this study and will act as the data controller for this study. This means that we are responsible for looking after your information and using it properly. The University will only keep your personal contact details until the interview is completed. This is in case we need to contact you. We will keep your name and contact details confidential and will not pass this information to unrelated parties.

All the information that is collected during the study about you is strictly confidential and your name and other personal details will not be shared in any final reports, presentations, articles or other outputs we create. If you were to share with us any information about poor practice or illegal activity or we are worried that someone is being harmed, then we would have to share this information with relevant people outside the study team. We would talk to you about this before we took any action.

The research student and the supervisory team will be able to access the data you provide (e.g. audio-recording of your interview) using secure University systems. As soon as audio-files have been transferred to the secure server they will be deleted from the recording device. The audio-files will be deleted from the secure server once the anonymised transcripts have been checked for accuracy and omissions. Your rights to access or remove your information are limited, as we need to manage your information in specific ways in order for the research to be reliable and accurate.

If you withdraw from the study, we will keep the information about you that we have already obtained, unless you tell us within 7 days of the interview that you do not want us to use it. To safeguard your rights, we will use the minimum personally-identifiable information possible.

Your consent form will be stored electronically and the original paper copies will be destroyed in an appropriate manner. Data and all appropriate documentation will be stored for a minimum of 10 years. After this, it will be destroyed securely, in line with the university protocols.

What will happen to the results of the study?

The results of the study will be shared across the NHS and we aim that they will inform treating children with a febrile illness. We plan to write articles and present the work to help us share our ideas with other professionals. Quotations of things you may say in the interview maybe included in the publications but these will be anonymous and you will not be identifiable from them.

Who is funding this study?

This study is part of a PhD research project. It is jointly funded by NIHR SPHR and NIHR ARC NWC.

Who has reviewed the study?

The study has been reviewed by the research study team, by an internal review at the University of Liverpool, and by the Health Research Authority, Research Ethics Service (committee details and numbers to be added).

Where can I find updates about the study?

This study is part of a wider research project. If you want to keep updated about the study, you can find information at https://arc-nwc.nihr.ac.uk/impact/our-phd-students-research/febrilestudy/

Who can I contact if we want to take part or want more information?

If you want to take part, or you would like us to tell you more about the project and answer your questions, please contact:

- Research Student, Courtney Franklin (073842 42202/Courtney.franklin@liverpool.ac.uk)

If you have any concerns about the research and wish to speak to someone independent, please contact sponsor@liverpool.ac.uk.

Thank you very much for your time in reading this information leaflet and for considering taking part in the study.

Doctor interview guide

CHildhood Inequalities in Liverpool in febriLe attendances to the Emergency Department

(CHILLED Study)

Interview Guide (HCPs)

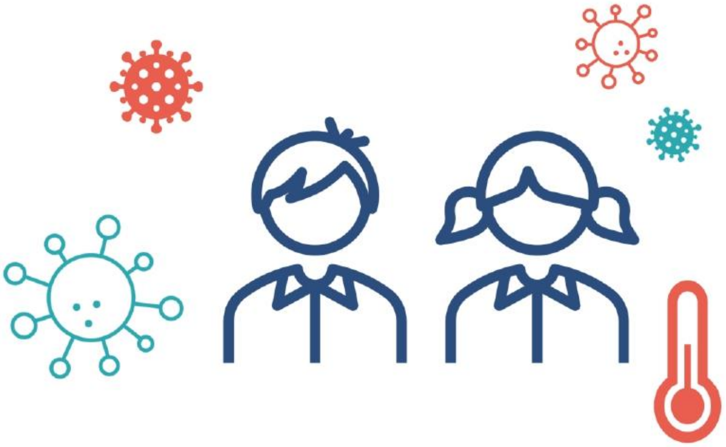

Study Team: Courtney Franklin, Prof David Taylor-Robinson, Prof Enitan Carrol and Prof Bernie Carter

1. Opening Section

The interviewer will summarise the study using the HCP information sheet, giving the participant an opportunity to ask questions, then gain consent (as per procedure). The interviewer will reassure the participant that there are no right or wrong answers and that they are just interested in their perceptions and experiences.

2. Main Section

The interviewer will invite the HCP to talk about their perceptions and experiences associated with treating febrile children at an Emergency Department (ED) in hospital. Using a mixture of open, and more directed questions, the researcher will engage the participant in conversation to elicit their perceptions of the reasons for ED presentation, and experiences associated with decisions to perform further diagnostic tests or prescribe antibiotics. A timeline will be used to help the participant to map their journey and explore who they had consulted for advice, how helpful this was, and what weight they placed on the advice. We intend to unpick issues related to HCP perceptions of parental concern in terms of fever and management of fever, decisions to perform further diagnostic tests or prescribe antibiotics and other treatment.

The interviewer following questions, and prompts will be used (although they will not necessarily be addressed in this order and the phrasing may change in response to individual contexts).

- Please tell me about a time/times you have treated a child who presented to the ED with a fever.
  - Think back to the most recent child/children they have cared for with a fever
  - How old were they?
  - What were they like? Did they seem unwell? Were they experiencing any other symptoms?
- Please tell me about your perception of why the parent/carer may have brought their child to the hospital.
  - Did they seem concerned? If yes, how seriously did you consider their concerns at this time? Do you know if they had attempted to be seen elsewhere (e.g at a GP)? Do you know if they followed advice to bring their child to hospital? Were you aware of any circumstances that may have led to their decision?
- Please tell me about your perception of the parent/carer's emotions at the time, and why you think they felt this way.
  - E.g. is it possible the parent/carer was stressed and concerned after having a long wait time before being seen? Do you think they had a good general understanding of fever?
- What happened when you saw the child?
  - Did you have any initial thoughts about why the child may be experiencing a fever?
  - What kind of questions did you ask the parent/carer?

    - Did the child have any tests? Why?
    - Was the child given any antibiotics? Did they receive any other medicines or treatments? Why?
- Please tell me about your perception of the emotions of the parent/carer during this time.
    - Do you think they were happy/unhappy with the treatment their child received?
    - Do you think they had come to hospital with certain expectations (e.g for their child to receive antibiotics)? How did you feel about this?
    - Did they express any concerns? What were they? How seriously did you acknowledge these concerns?
- Please tell me about the discharge of the patient.
    - What was the diagnosis?
    - Did you provide extra information for the parent/carer when they left?
    - Do you think the parent/carer felt more confident about managing their child at home after you had spoken to them?
- If you were to experience this again in the future, is there anything you would do differently?
    - Is there anything that you think could have made the experience easier?

3. Closing Section

The interviewer will thank the participant, checking that they are okay and ask if they have any other questions.
