# Supplementary material for: Coming in hot: a qualitative investigation into perceptions of parents and doctors of reasons for the presentation of children with fever to the emergency department in England
Source: BMJ Paediatr Open. 2024 Dec 24;8(1):e003039. doi: 10.1136/bmjpo-2024-003039 (PMC11683883; doi:10.1136/bmjpo-2024-003039)
Supplement: Supplementary file 2 [file bmjpo-2024-003039.reviewer_comments.pdf]

PEER REVIEW HISTORY

BMJ Paediatrics Open publishes all reviews undertaken for accepted manuscripts. Reviewers are asked to complete a checklist review form and are provided with free text boxes to elaborate on their assessment. These free text comments are reproduced below.

ARTICLE DETAILS

**TITLE (PROVISIONAL)**	Coming in hot: A qualitative investigation into perceptions of parents and doctors of reasons for presentation of children with fever to the Emergency Department in England.
**AUTHORS**	franklin, courtney; Taylor-Robinson, David; Carrol, Enitan; Moran, Paul; Carter, Bernie

VERSION 1 - REVIEW

**REVIEWER NAME**	***Damian Roland***
**REVIEWER AFFILIATION**	University of Leicester, Health Sciences
**REVIEWER CONFLICT OF INTEREST**	
**DATE REVIEW RETURNED**	17-Oct-2024

**GENERAL COMMENTS**	Thank you for addressing comments from the previous review

**REVIEWER NAME**	***Shammi Ramlakhan***
**REVIEWER AFFILIATION**	Sheffield Children's NHS Foundation Trust, Emergency Department
**REVIEWER CONFLICT OF INTEREST**	
**DATE REVIEW RETURNED**	24-Oct-2024

**GENERAL COMMENTS**	Abstract:<br>The last phrase in the conclusion can be removed ' to allow those that really<br>need it, get the care efficiently' - this is an oversimplification, and not really what your study is about. Also p9, line 52<br><br>Introduction:<br>It is unclear what the first 3 sentences add in the context of the paper.<br>How does an ED admission and attendance differ - this nomenclature should be consistent throughout (use ED attendance).<br>The statement about inequalities, while possibly important means little without contextualisation for ED attendance with fever. Suggest remove.<br><br>It would be more helpful to know what proportion of ED attendances are for febrile illness rather than it being part of the top 10. You can perhaps get this information from ECDS or similar repositories.<br>In addition, you mention serious illness, but isn't this more about self limiting/low acuity illness? What proportion of those attending with a febrile illness actually have a serious illness or require ED input/attendance/hospital admission?

	Other authors have suggested that parents attend ED with lower acuity conditions (https://pubmed.ncbi.nlm.nih.gov/31116119/) like fever for reasons similar to those you have hypothesised/demonstrated. This may help with the rationale for the study as this could be clearer in the Introduction - specifically relating to perceptions of urgency/severity of a fever; uncertainty regarding their own assessment of the severity/urgency of a fever; the possibility that managing their child's fever may require resources available solely at the ED (rather than say at home or primary care); and (lack of) awareness of alternative services.<br>In addition, previous utilisation of these alternative services for fever, which lead to them then being referred to ED, also influences future decision making.<br><br>p3, line 59; p8, line 41- It is a stretch to imply that the aim is to be representative of England. It samples almost exclusively from NW England...<br><br>Methods:<br>The parent sample is from those attending ED from January 2015, whereas the interviews were between June 2022-Jan 2023. Are you confident that parents will recall their perceptions, decision making and thought process 7 years later?<br><br>What is venue-based sampling? (is this just recruiting from ED?)<br>If you wanted to truly recruit from 'areas of higher disadvantage' wouldn't purposive sampling been preferable than convenience sampling? You have not presented IMD or other recognised measures of deprivation in the participant characteristics, so mentioning this as a rationale for sampling means that you did not adhere to this strategy or it was added post-hoc.<br><br>For software etc (Zoom, NViVo and so on), it is standard to include (company, location)<br><br>p5, line 8 - 'Ethical approval was granted by...' rather than 'Ethics was approved...'<br><br>Discussion:<br>p8, line 57 - It is fair to say that risk aversion also exists with parents and other non-community clinicians/HCPs (https://pubmed.ncbi.nlm.nih.gov/31116119/) and drives health seeking behaviour (in terms of ED attendance).<br><br>p9, line 15 - Are you advocating that parents should all have a thermometer and measure their child's temperature to aid decision making? This feels counterintuitive to the idea of reassurance and a holistic approach to a parent managing a hot child. The 'definition' of a fever suggested by the (?junior doctor) is not that straightforward and the actual number is variable (even the NHS scot website cannot decide between 37.8 and 38!) - nevertheless, outside of specific patient groups, it is perhaps unhelpful to encourage fixation on the specific temperature as this will likely just result in conflicting information.<br>This is seen with febrile convulsions, where parents fixate on avoiding a 'fever' and feel guilt if the child inevitably has a seizure - we know that febrile seizures are dependent upon a threshold temperature which varies from one child to another and with age and other factors.

	p9, line 55 - Not sure what this first sentence adds - suggest remove.

VERSION 1 – AUTHOR RESPONSE

Reviewer 1:
Dr. Damian Roland, University of Leicester, University Hospitals of Leicester NHS Trust

1) Thank you for addressing comments from the previous review
Response: Thank you.

Reviewer: 2
Dr. Shammi Ramlakhan, Sheffield Children's NHS Foundation Trust

2) Abstract:
The last phrase in the conclusion can be removed ' to allow those that really
need it, get the care efficiently' - this is an oversimplification, and not really what your study is about.
Also p9, line 52

Response: Thank you, this has been removed.

3) Introduction:
It is unclear what the first 3 sentences add in the context of the paper.

Response: Thank you, we have clarified this by adding a sentence at the end of the paragraph:

“Previously, UK emergency department (ED) attendances increased year on year [1] with a similar trend for admissions from the ED, posing a huge burden on the NHS financially. The ten most common presenting problems in children account for around 85% of child ED attendances in the UK [4, 5]. Febrile illness accounts for around 14% of these consultations[3]. There are many causes of fever, but most are due to self-limiting illness. Therefore, there remains a need to establish the reasons behind emergency presentations for febrile children.”

4) How does an ED admission and attendance differ - this nomenclature should be consistent throughout (use ED attendance).

Response: Thank you, we have standardised the language throughout to ED attendance, and also refer to hospital admission from ED. Whilst this study investigates reasons for ED presentations (attendances), it also allowed participants to talk openly about their experiences throughout their healthcare journey, these have included admission from the ED to hospital. Specifically, these two terms differ as a child can present to the ED but may not be admitted from the ED to hospital. The introduction also provides context of rising attendances and admissions from the ED, as well as previous literature exploring both attendances and admissions from the ED. I have made some minor adjustments to phrases within the introduction for clarity (found in the marked copy).

5) The statement about inequalities, while possibly important means little without contextualisation for ED attendance with fever. Suggest remove.

Response: This has been removed, thank you.

6) It would be more helpful to know what proportion of ED attendances are for febrile illness rather than it being part of the top 10. You can perhaps get this information from ECDS or similar repositories.

Response: Thank you, I have added additional information regarding current cases of febrile emergency presentations (“Febrile illness accounts for around 14% of these consultations “). However, since febrile illness is a presenting problem and not a disease (and therefore does not have a specific diagnosis code), there is extremely limited quantitative research examining emergency health service longitudinal trends for these presentations.

7) In addition, you mention serious illness, but isn't this more about self limiting/low acuity illness? What proportion of those attending with a febrile illness actually have a serious illness or require ED input/attendance/hospital admission?

Response: Thank you for your comments. Serious illness was mentioned, as existing literature has focused on this, but not specifically for all febrile cases (of which many are self-limiting). This was therefore an identified gap in evidence that this paper sought to expand upon. I have amended paragraph 3 to better explain this:

“Fragmented services can impact parents’ experiences when navigating healthcare for their child and cause confusion about where to have their health needs met[13-15]. Fragmented services and parents’ problems interpreting symptoms are core modifiable factors influencing the timing of attendance and admission to hospital for children with serious infectious illness [16]. Little evidence exists on these pathways for fever (of which many cases could be self-limiting), or doctors’ perceptions of why parents present their child to the ED and how consideration of this can affect the child’s treatment[17]. We aimed to understand parental and doctor perceptions of the reasons for ED presentation for children with fever in England.”

8) Other authors have suggested that parents attend ED with lower acuity conditions (https://pubmed.ncbi.nlm.nih.gov/31116119/) like fever for reasons similar to those you have hypothesised/demonstrated. This may help with the rationale for the study as this could be clearer in the Introduction - specifically relating to perceptions of urgency/severity of a fever; uncertainty regarding their own assessment of the severity/urgency of a fever; the possibility that managing their child's fever may require resources available solely at the ED (rather than say at home or primary care); and (lack of) awareness of alternative services.
In addition, previous utilisation of these alternative services for fever, which lead to them then being referred to ED, also influences future decision making.

Response: Thanks, we have added this to the introduction as suggested:

“Parental uncertainty and low risk tolerance have been previously identified as drivers for ED attendance for conditions suitable for management in less acute settings (McLauchlan et al., 2020). Therefore, whilst increased admissions may not be attributed to increased severity of disease, increased perceived severity by parents may increase ED attendances and influence risk averse behaviours among parents and doctors.”

9) p3, line 59; p8, line 41- It is a stretch to imply that the aim is to be representative of England. It samples almost exclusively from NW England...

Response: Thanks, we have made this clearer in the limitations:

“Most participants were located in the Northwest of England. Further qualitative work in other populations across England would improve the generalisability of results and help to investigate parental reasons for ED attendance for children with febrile illness.”

10) Methods:
The parent sample is from those attending ED from January 2015, whereas the interviews were between June 2022-Jan 2023. Are you confident that parents will recall their perceptions, decision making and thought process 7 years later?

Response: Thank you. This was thoroughly considered by the research team. However, the experience of bringing a child into A&E can be traumatic and will most definitely be a memorable one, especially during the lockdown. Therefore, we decided this would not affect the quality of recalling such an experience. The specified timeframe was only detailed within documents for ethics approval. Therefore, when a parent self-referred to this study, it was considered that they deemed their experience sufficiently memorable to discuss their experience in detail.

We have made a note about this in the limitations section:

“For some of the participants there was a time gap between admission and interview which may impact recall of parent perceptions and decision making. However, this evidence points to the emotional significance of ED visits [41], which may make them more memorable over time. Additionally, using probing questions to cue specific aspects of their experience helped to stimulate responses”.

11) What is venue-based sampling? (is this just recruiting from ED?)
If you wanted to truly recruit from 'areas of higher disadvantage' wouldn't purposive sampling been preferable than convenience sampling? You have not presented IMD or other recognised measures of deprivation in the participant characteristics, so mentioning this as a rationale for sampling means that you did not adhere to this strategy or it was added post-hoc.

Response: Thank you for your comment, it is clear this needs further clarity in the manuscript.

The following has been added to the methods subsection of the manuscript to address this:

“Convenience and venue-based sampling facilitated timely access of potential participants and encouraged uptake of participants from areas of higher disadvantage (the NorthWest of England). Convenience sampling was the most accessible form of sampling and allowed all eligible, consenting members take part in the study. Consideration was further made to incorporate a sampling frame for homogeneous convenience sampling. This has previously been done by intentionally constraining the sampling frame with respect to sociodemographic background [19]. Upon advisement from our public advisor, we did not collect socio-economic information from prospective or actual participants, as it was felt this could be perceived as intrusive and could deter participants. Instead, we used a form of venue-based sampling, identifying locations where the target population may gather, randomly selecting and visiting those locations, and systematically intercepting potential consenting participants [20]. Recruitment was intentionally focused on Liverpool and the surrounding areas in Northwest England to capture perceptions within an area of historical and current disadvantage. This allowed sampling to be targeted to parents in areas of a higher deprivation who may have been missed using other sampling strategies. This was done by contacting nursery schools, primary schools and children’s centres to help distribute recruitment adverts, as well as focusing social media advertisement within these targeted areas”.

12) For software etc (Zoom, NViVo and so on), it is standard to include (company, location)

Response: Thank you, this has now been included.

13) p5, line 8 - 'Ethical approval was granted by...' rather than 'Ethics was approved...'

Response: This has been amended.

14) Discussion:
p8, line 57 - It is fair to say that risk aversion also exists with parents and other non-community clinicians/HCPs (https://pubmed.ncbi.nlm.nih.gov/31116119/) and drives health seeking behaviour (in terms of ED attendance).

Response: Agreed. This line has been amended accordingly.

“ED doctors perceived fever phobia and risk aversion existed among parents and HCPs (in both community and non-community settings), noting this encouraged emergency care use[12, 14].”

15) p9, line 15 - Are you advocating that parents should all have a thermometer and measure their child's temperature to aid decision making? This feels counterintuitive to the idea of reassurance and a holistic approach to a parent managing a hot child. The 'definition' of a fever suggested by the (?junior doctor) is not that straightforward and the actual number is variable (even the NHS scot website cannot decide between 37.8 and 38!) - nevertheless, outside of specific patient groups, it is perhaps unhelpful to encourage fixation on the specific temperature as this will likely just result in conflicting information. This is seen with febrile convulsions, where parents fixate on avoiding a 'fever' and feel guilt if the child inevitably has a seizure - we know that febrile seizures are dependent upon a threshold temperature which varies from one child to another and with age and other factors.

Response: Thanks. We don’t advocate for focusing upon a specific temperature. Instead, we highlight the need for consistent information. As you also suggest, there is no consistent definition for what constitutes a fever. There is also a lack of consistent information regarding warning signs for

serious infection, or other conditions that require emergency care. To ensure it is clear we are not advocating for fixation on temperature thresholds, we have amended the statement as follows:

“HCPs need to provide clear consistent fever-related information (e.g., the warning signs which would justify emergency attendance). Eliminating conflicting information would support parental decision-making, encourage trust in HCPs[25], and prevent selective use of emergency services.”

16) p9, line 55 - Not sure what this first sentence adds - suggest remove.

Response: Removed.
